# Investigation of the host transcriptional response to intracellular bacterial infection using *Dictyostelium discoideum* as a host model

**DOI:** 10.1186/s12864-019-6269-x

**Published:** 2019-12-10

**Authors:** Jonas Kjellin, Maria Pränting, Frauke Bach, Roshan Vaid, Bart Edelbroek, Zhiru Li, Marc P. Hoeppner, Manfred Grabherr, Ralph R. Isberg, Monica Hagedorn, Fredrik Söderbom

**Affiliations:** 1 Department of Cell and Molecular Biology, Uppsala University, Uppsala, Sweden; 2 Present Address: ReAct – Action on Antibiotic Resistance, Department of Medical Sciences, Uppsala University, Uppsala, Sweden; 3 Section Parasitology, Bernhard Nocht Institute for Tropical Medicine, Hamburg, Germany; 4 Present Address: Department of Stem Cell Transplantation, University Medical Center Hamburg-Eppendorf, Hamburg, Germany; 5 Department of Molecular Biosciences, The Wenner-Gren Institute, Stockholm University, Stockholm, Sweden; 6 Department of Molecular Biology and Microbiology, Tufts University School of Medicine, Boston, MA 02111 USA; 7 Present Address: New England Biolabs, Ipswich, MA USA; 8 Department of Medical Biochemistry and Microbiology, Uppsala University, Uppsala, Sweden; 9 Institute of Clinical Molecular Biology, Kiel University, University Hospital Schleswig-Holstein, Kiel, Germany; 10 Life Sciences and Chemistry, Jacobs University Bremen gGmbH, Group Ribogenetics, Bremen, Germany

**Keywords:** Host-pathogen, Infection, High-throughput sequencing, Mycobacteria, Legionella, *Dictyostelium discoideum*, Macrophage, Infection model, Pathogenic bacteria, Intracellular pathogen

## Abstract

**Background:**

During infection by intracellular pathogens, a highly complex interplay occurs between the infected cell trying to degrade the invader and the pathogen which actively manipulates the host cell to enable survival and proliferation. Many intracellular pathogens pose important threats to human health and major efforts have been undertaken to better understand the host-pathogen interactions that eventually determine the outcome of the infection. Over the last decades, the unicellular eukaryote *Dictyostelium discoideum* has become an established infection model, serving as a surrogate macrophage that can be infected with a wide range of intracellular pathogens. In this study, we use high-throughput RNA-sequencing to analyze the transcriptional response of *D. discoideum* when infected with *Mycobacterium marinum* and *Legionella pneumophila*. The results were compared to available data from human macrophages*.*

**Results:**

The majority of the transcriptional regulation triggered by the two pathogens was found to be unique for each bacterial challenge. Hallmark transcriptional signatures were identified for each infection, e.g. induction of endosomal sorting complexes required for transport (ESCRT) and autophagy genes in response to *M. marinum* and inhibition of genes associated with the translation machinery and energy metabolism in response to *L. pneumophila*. However, a common response to the pathogenic bacteria was also identified, which was not induced by non-pathogenic food bacteria. Finally, comparison with available data sets of regulation in human monocyte derived macrophages shows that the elicited response in *D. discoideum* is in many aspects similar to what has been observed in human immune cells in response to *Mycobacterium tuberculosis* and *L. pneumophila.*

**Conclusions:**

Our study presents high-throughput characterization of *D. discoideum* transcriptional response to intracellular pathogens using RNA-seq. We demonstrate that the transcriptional response is in essence distinct to each pathogen and that in many cases, the corresponding regulation is recapitulated in human macrophages after infection by mycobacteria and *L. pneumophila*. This indicates that host-pathogen interactions are evolutionary conserved, derived from the early interactions between free-living phagocytic cells and bacteria. Taken together, our results strengthen the use of *D. discoideum* as a general infection model.

## Background

In order to establish an infection, intracellular bacterial pathogens have to subvert the degradation by the host cell as well as establish a suitable niche for proliferation. At the same time, the infected host turns on defense mechanisms to clear the infection. This leads to a series of complex and dynamic host-pathogen interactions that eventually will determine the outcome of the infection.

*Dictyostelium discoideum*, a social amoeba*,* is a professional phagocyte that can rapidly ingest and degrade bacteria for nutrients. However, several bacterial pathogens have been shown to avoid degradation by the amoeba and establish a replicative niche by manipulating the hosts intracellular machinery. The processes used by the bacteria to establish an infection in *D. discoideum,* are in many aspects very similar to the infectious route in mammalian macrophages [1]. For these reasons, *D. discoideum* has over the past decades emerged as a valuable model system to study the basic interactions between a host cell and a wide range of intracellular pathogens, e.g. *Legionella pneumophila*, different mycobacterial species, and *Francisella noatunensis* (reviewed in [2–4]).

The genus *Mycobacterium* comprises several bacterial species of which many are pathogenic to humans. The most well-known of these is the causative agent of tuberculosis (TB), *Mycobacterium tuberculosis*, which is among the top ten causes of death in the world [5]. In addition, approximately one-quarter of the world’s population carries a latent TB infection which may reactivate and spread at a later time [5]. *Mycobacterium marinum* is a close genetic relative to *M. tuberculosis* and the key virulence factors are conserved between the two species, such as five type VII secretion systems, ESX-1 to ESX-5 [6]. The disease progression in the natural hosts of *M. marinum*, e.g. fish and amphibians, is analogous to the disease progression of *M. tuberculosis* in humans. *M. marinum* can induce granulomatous lesions, as well as develop into a latent disease, which are both hallmark traits of TB [7]. In addition, the intracellular route shortly after uptake of *M. marinum* is similar to that of *M. tuberculosis*. Both pathogens avoid degradation by arresting phagosome maturation leading to the establishment of the mycobacteria containing vacuole (MCV) and subsequent escape to the cytosol of the host [8–11]. As a unicellular model, *D. discoideum* can mainly be used to study the early interaction between the pathogen and the host, i.e. before the formation of granulomas and establishment of latent infection which occurs in more complex organisms. Overall, an *M. marinum* infection of a *D. discoideum* culture can last up to 37 h [12]. However, the pathogen needs to take action almost immediately after entry into the host cell in order to survive since bacteria are usually killed within minutes after uptake [13]. *M. marinum* avoids degradation by active manipulation of several host factors, e.g. GTPases [12, 14, 15] and autophagic machinery components [15], in order to prevent normal phagosome maturation and to establish a replication permissive environment (MCV) within the host [3]. This infection phase, under which little or no proliferation of *M. marinum* occurs, lasts up to approximately 12 h post infection (hpi) and is followed by an enhanced proliferation phase (~ 12–37 hpi) after which bacterial proliferation is arrested due to bacterial death or release from the host cell (reviewed in [3]).

In contrast to *M. tuberculosis, L. pneumophila* is often considered to be an accidental pathogen to humans and infection in human generally constitutes a dead end for the bacteria [16]. In most cases, *L. pneumophila* infection spreads via aerosols from water reservoirs and causes a special type of pneumonia, Legionnaires’ disease, which can be fatal [16]. In nature, several amoebae, such as *Acanthamoeba* spp*.,* are reservoirs for the bacteria and are considered to be important drivers for the evolution of bacterial pathogenicity [17]. In order to survive and proliferate within a host cell, in macrophages and amoeba alike, the pathogen actively manipulates the host cell by translocating more than 300 effectors via the Dot/Icm type IVb translocation/secretion system (T4SS). These secreted virulence factors prevent for example lysosome fusion with the pathogen containing vacuole and allow the bacterium to establish the replicative *Legionella-*containing vacuole (LCV) (reviewed in [18]).

Despite extensive research on host-pathogen interactions during infection with both mycobacteria and *L. pneumophila*, much is still unknown about the early critical steps in which the pathogen needs to actively manipulate the host cell in order to survive and create a replication permissive environment. In this study, we investigated the transcriptional changes early after infection by *M. marinum* and *L. pneumophila*, respectively, using high-throughput RNA-sequencing (RNA-seq). Distinct, as well as common transcriptional changes were detected in the host in response to the pathogens*.* Infection by *M. marinum* affected processes such as intracellular trafficking, membrane trafficking, and autophagy, illustrated by differential expression of genes encoding e.g. GTP-binding proteins and the ESCRT machinery. In contrast, in *L. pneumophila* infected cells, genes were regulated that are primarily involved in host growth e.g. ribosome biogenesis and energy metabolism, as well as genes central to the production of reactive oxygen species (ROS), important for killing pathogens. Importantly, the transcriptional responses in *D. discoideum* upon infection by the pathogens are in many aspects similar to the regulatory changes observed in human macrophages infected with *M. tuberculosis* or *L. pneumophila* [19, 20], strengthening the role of *D. discoideum* as a model for cellular responses during uptake and early interaction with different pathogenic bacteria.

## Results

### High-throughput sequencing of *D. discoideum* cells infected with *M. marinum* and *L. pneumophila*

**Fig. 1:**
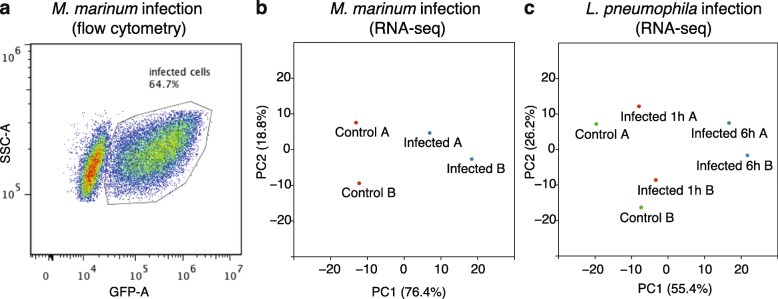
RNA-seq sample preparation and quality control. **a** Flow cytometry analysis of proportion of *D. discoideum* cells infected with *M. marinum*. **b**, **c** Principal component analysis of RNA-seq data from *D. discoideum* cells infected with *M. marinum* or *L. pneumophila* (1 h: 1 hpi; 6 h: 6 hpi) as well as non-infected (Control) cells. A and B: biological duplicates

In order to characterize the early transcriptional regulation of host genes after infection by *M. marinum* and *L. pneumophila* respectively, we performed high-throughput sequencing of poly(A) enriched RNA from infected and non-infected (control) cells. To obtain RNA for transcriptional studies of *M. marinum* infected *D. discoideum*, we used a high multiplicity of infection (MOI of 200) in order to acquire a strong and synchronized transcriptional signature of infected cells already 2.5 h post infection (hpi). Furthermore, we aimed for similar proportions of infected cells (around 60%) as for the *L. pneumophila* infection (see below). Flow cytometry analysis revealed that approximately 65% of the *D. discoideum* cells carried *M. marinum* at this time point (Fig. 1a). Cell viability could be a concern at this high MOI. Hence, to assay cell death during infection, we challenged *D. discoideum* cells with *M. marinum* as described above but with different MOI of *M. marinum*. The fraction of dead cells were assayed by propidium iodide staining followed by flow cytometry [21]. The results clearly showed that while the proportion of infected cell increased with higher MOI, the cell viability was not affected to great extent since the fraction of dead cells only increased from ~ 2% for uninfected *D. discoideum* cells up to ~ 8% at MOI 200 (Additional file 1: Figure S1).

The early host response to *L. pneumophila* infection has previously been investigated in *D. discoideum* using microarray transcriptome analyses [22, 23]. However, one limitation of these studies is that the microarrays only covered approximately 5400 [22] or 8600 [23, 24] genes out of more than 12,200 protein coding genes in *D. discoideum* [25]. Therefore, we used high-throughput RNA-seq to further investigate the transcriptional response to *L. pneumophila* infection. This also allowed us to do a global comparison of regulated genes triggered by *M. marinum* and *L. pneumophila* infections respectively. RNA-seq was performed on RNA collected 1 and 6 h after *L. pneumophila* infection as well as on RNA prepared from non-infected *D. discoideum* cells. Notably, the RNA used for our RNA-seq study had previously been isolated by Li and coworkers who performed microarray analysis on the same batch of RNA isolated from non-infected cells and cells collected 6 h post *L. pneumophila* infection [23]. Hence, this also allowed us to perform an evaluation of the two different methods: microarray versus high-throughput RNA-seq analysis (see below).

Each high-throughput sequencing yielded a mean of 18.6 and 18.8 million reads from *D. discoideum* non-infected cells or cells infected with *M. marinum* respectively, that mapped to the genome after quality control and filtering steps. The same analyses for *L. pneumophila* infected and non-infected cells yielded 11.4 and 11.5 million reads.

Principal component (PC) analyses were performed for each type of infection (biological replicates), including their respective non-infected controls (separate for each infection experiment). The result clearly showed that the infected and non-infected samples separated along principal component 1 (PC1) in response to both *M. marinum* and *L. pneumophila* (Fig. 1b, c).

### Large transcriptional responses early after infection

**Fig. 2:**
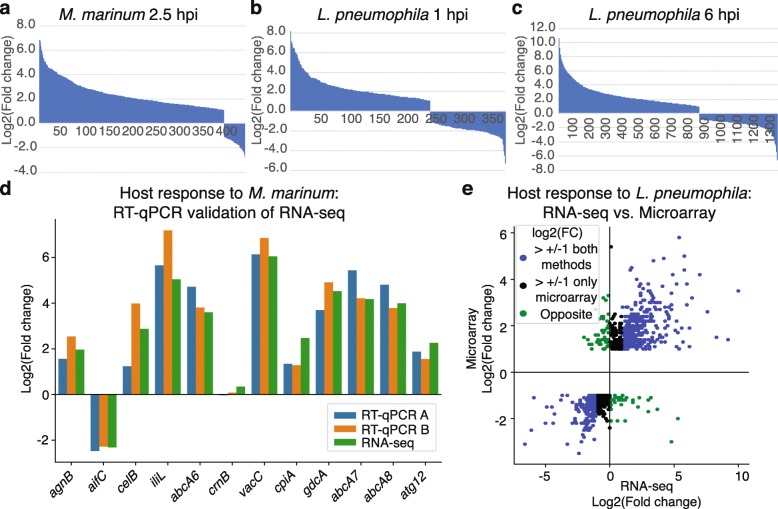
Differential gene expression in response to *M. marinum* and *L. pneumophila* infection. **a**–**c** Summary of gene regulation in *D. discoideum* in response to separate infections with *M. marinum* **a** and *L. pneumophila* 1 hpi and 6 hpi **b**, **c**, respectively. X-axes represent number of genes (FDR < 0.05) and Y-axes display the regulation of genes in comparison to non-infected controls. **d** RT-qPCR validation of differential expression of genes in response to *M. marinum* infection*.* RT-qPCR was performed on RNA from the same two infection experiments used for RNA-seq, including respective non-infected controls for differential expression analyses. **e** Comparison of gene regulation detected by microarray [23] with the corresponding regulation determined with RNA-seq for *L. pneumophila* infected cells. Marked in *blue*: genes with log2(fold change) bigger than 1 or smaller than − 1 according to both methods; Marked in *black*: genes with log2(fold change) bigger than 1 or smaller than − 1 according to microarray but not RNA-seq; Marked in *green*: genes showing opposite regulation according to the different methods. Note that the ranges for the two axes differ

Differential expression analysis of infected versus non-infected samples was performed for each infection, *M. marinum* or *L. pneumophila*, using DESeq2 [26] and genes with a false discovery rate (FDR) < 0.05 were considered to be differentially regulated. For both *M. marinum* 2.5 hpi and *L. pneumophila* one hpi, approximately 400 genes were found to be differentially regulated while more than 1300 genes were differentially expressed 6 h post *L. pneumophila* infection (Additional files 2 and 3). In cells infected with *M. marinum*, the great majority of the regulated genes showed increased expression while a more even distribution between up- and down-regulated genes was observed for *L. pneumophila* infected *D. discoideum* cells (Fig. 2a–c). Separate reverse transcription quantitative PCR (RT-qPCR) was performed on the two RNA-seq replicates to validate the regulation of 12 genes that were up-, down-, and non-regulated in the RNA-seq analysis of *M. marinum* infected cells and all tested genes showed comparable levels of regulation with both methods (Fig. 2d). The infection was repeated three times and RT-qPCR confirmed the differential expression induced by *M. marinum* infection, indicating a robust and repeatable gene expression response. This was also apparent when the new RT-qPCR data was compared to the RNA-seq analyses (Additional file 1: Figure S2a-c).

In summary, high-throughput sequencing of RNA from *D. discoideum* infected by *M. marinum* and *L. pneumophila* shows that many genes are differentially expressed already at early time points after uptake of either bacterium. In particular, a dramatic response is set off 6 h after infection with *L. pneumophila* at which time more than 1300 genes are differentially expressed.

### High throughput RNA-seq and microarray analyses yield similar results

Next we compared the gene regulation 6 h after *L. pneumophila* infection detected by RNA-seq with the previously reported differential gene expression identified by microarray, using the same batch of RNA (Additional file 3) [23]. The RNA-seq analysis, representing all ~ 12,200 genes in *D. discoideum*, showed differential regulation of 1300 genes (FDR < 0.05, see above), while ~ 900 of the 8600 genes on the microarray were reported as differentially expressed (*p*-value < 0.05 and log2(FC) > 1 or < − 1) [23]. In order to compare the result from the two methods, we compared the fold changes for the genes identified as significantly differentially regulated by microarray [23] with the changes for the same genes in the RNA-seq data. More than 60% showed similar regulation with a log2(FC) > 1 or < − 1 also in the RNA-seq analysis (Fig. 2e, marked in blue), while approximately 30% showed similar but weaker regulation, including some that appeared unregulated in the RNA-seq analyses (Fig. 2e, marked in black). Less than 9% showed opposite regulation between the two methods (Fig. 2e, marked in green). When we compared the regulation of differentially expressed genes as defined by RNA-seq (FDR < 0.05) to the regulated genes on the microarray (as defined above), more than 99% (446 out of 450) genes showed similar regulation (Additional file 1: Figure S3, Additional file 3).

Notably, of the 1300 genes identified as differentially expressed by RNA-seq, ~ 600 genes had not previously been reported as associated with transcriptional response upon *L. pneumophila* infection of *D. discoideum*. In part, this can be explained by the fact that more than 260 of these genes were not included in the microarray design.

Taken together, the RNA-seq and microarray analyses give highly similar results when the host gene expression response to *L. pneumophila* is compared, which is in line with previously reported comparisons of microarray and RNA-seq transcriptomics data [27].

### *D. discoideum* response to *M. marinum* is enriched for genes involved in intracellular trafficking, autophagy and phagosome maturation

In order to interpret the transcriptional response triggered by *M. marinum*, we performed gene ontology (GO) term enrichment analysis for up- and down-regulated genes, respectively. Full list of enriched GO-terms and associated genes are available in Additional file 4. Additional results and key genes involved in the different processes can be found in Additional file 1: Additional results and Table S1.

#### GTP-binding proteins and actin

**Fig. 3:**
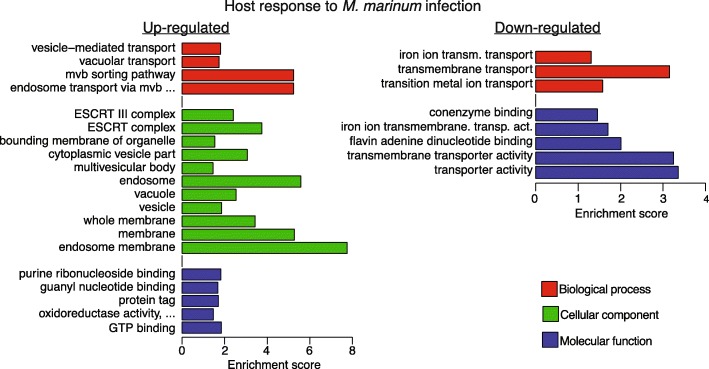
Gene ontology enrichment analysis for regulated *D. discoideum* genes in response to *M. marinum*. The graphs show a subset of the enriched terms for the up- and down-regulated genes, respectively. Names of some GO terms are abbreviated due to size limitations. The full set of enriched terms, including full names, and associated genes, is presented in Additional file 4. Enrichment score equals Log(1/corrected *P*-value)

Among the up-regulated genes we detected an enrichment of genes coding for GTP binding proteins (Fig. 3, Additional file 4). The majority of these genes are small GTPases belonging to the Ras superfamily, which are known to be important regulators involved in a wide range of biological processes (reviewed in [28]). In our data, several up-regulated genes belong to the Rab family GTPases, whose members are mainly involved in the regulation of intracellular vesicular transport by e.g. enabling vesicle formation and facilitating vesicle fusion [28]. We also detected increased gene expression of several members of Ras and Rho family GTPases, important regulators of e.g. gene expression [28]. Rho family GTPases are also involved in regulating actin reorganization, which is critical for both phagocytosis [29] and subsequent phagosome maturation [30]. The effect on actin dynamics was underscored by the up-regulation of genes for dynamin GTPases and the increased expression of several actin and actin binding protein genes (Additional file 2).

#### ESCRT and membranes

GO-term enrichment analysis revealed that genes associated with Endosomal Sorting Complexes Required for Transport (ESCRT) were enriched among the up-regulated genes in response to *M. marinum* infection. In macrophages, *M. tuberculosis* interfere with the ESCRT machinery, which in turn prevents normal phagosome maturation [31, 32]. In *D. discoideum*, three of the main complexes, ESCRT-I, −II and -III, are well conserved [33] and the majority of the ESCRT-I and ESCRT-III associated genes were up-regulated in response to *M. marinum* infection. The genes for ESCRT-II, which is not essential for the function of the ESCRT machinery [34], were unaffected. In addition, we detected up-regulation of the ESCRT-associated genes involved in e.g. recruitment of ESCRT-I components to cytoplasmic membranes.

#### Autophagy

The ESCRT machinery is also required for macroautophagy, hereafter referred to as autophagy, however its exact role in this process remains to be determined [35]. Although autophagy was not detected as an enriched GO-term in itself, many genes associated with this process were found in several other enriched GO terms, e.g. membrane, vacuole and protein tag (Additional file 4). The autophagic machinery is involved in several steps of the infectious route of *M. marinum* in *D. discoideum,* from MCV rupture to the egress of the bacteria through non-lytic ejection [9, 12, 15, 36]. This also applies to human cells where *M*. *tuberculosis* manipulates the autophagic machinery to ensure survival within the host [37, 38].

Most of the regulated genes identified with RNA-seq that are associated with autophagy and their products have previously been individually characterized during *M. marinum* infection in *D. discoideum* [9, 12, 15, 36]. However, our data revealed increased expression levels of *atg5, atg12* and *atg18,* which previously have not been associated with *M. marinum* infection, as well as five ubiquitin genes. The Atg5-Atg12 complex is involved in phagophore membrane elongation [39]. Functional autophagy also relies on receptors which bridge the connection between the phagophore and the cargo marked for degradation [39]. Our data showed that two of the three proposed autophagy receptors in *D. discoideum* [39] were upregulated upon *M. marinum* infection.

### Genes for transmembrane transporters are downregulated during *M. marinum* infection

Although differential expression analysis showed that the majority of the affected genes were upregulated in *D. discoideum* cells infected by *M. marinum*, a fraction (9%) displayed reduced expression. These genes were mainly enriched for GO-terms involved in transmembrane transport (Fig. 3, Additional file 4) and included genes for ATP binding cassette (ABC) G family transporters and iron transporters (orthologues to natural resistance associated to macrophages 1 (*nramp1*) and mitoferrin (*mcfF*)).

### Transcriptional response to *L. pneumophila* infection is established already 1 h post infection

In order to characterize the dynamics of the transcriptional response after *L. pneumophila* infection, we compared the regulation at 1 and 6 h post infection. Of the 380 differentially regulated genes identified at 1 h post infection, 80% was differentially expressed also at 6 h post infection, indicating that the majority of the regulation induced 1 h post infection is maintained at least until 6 h post infection (Additional file 1: Figure S4a and marked in red in Additional file 1: Figure S4b, Additional file 3). However, a considerably larger response was detected at the later time point (1331 vs 380 regulated genes) (Additional file 1: Figure S4a). Interestingly, more than 95% of the genes affected at 6 h post infection (FDR < 0.05) showed similar regulation at the earlier time point when a less stringent cut off was used (cut off = log2(fold change) +/− 0.5) (Additional file 1: red and black marking in Figure S4b). This indicates that almost the entire response detected at 6 h post infection is induced already after 1 h but becomes more pronounced as infection progresses. Some of the differentially regulated genes are discussed below and an extended description, including gene names, can be found in Additional file 1: Additional results and Table S1.

### *L. pneumophila* infection induces expression of genes related to defense responses in *D. discoideum*

**Fig. 4:**
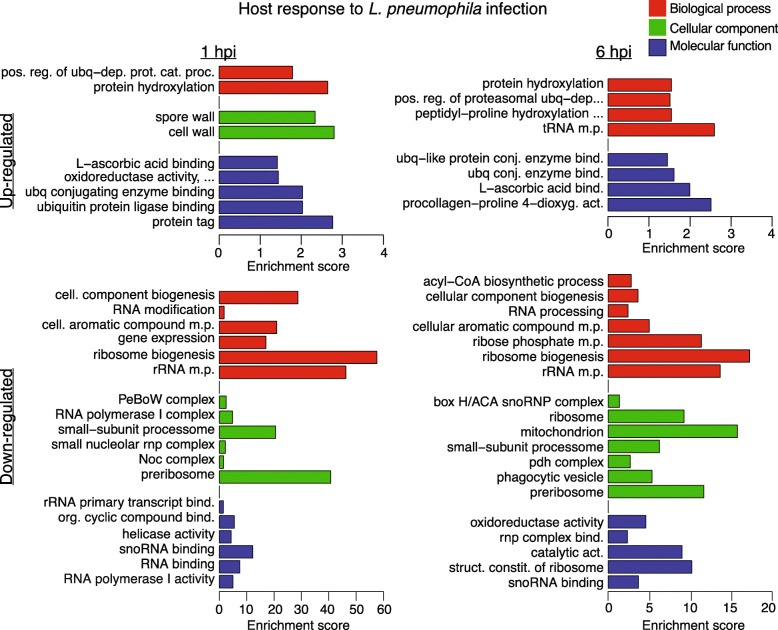
Gene ontology enrichment analysis for regulated *D. discoideum* genes in response to *L. pneumophila*. The graphs show a subset of the enriched terms for the up- and down-regulated genes at 1 and 6 hpi. Names of some GO terms are abbreviated due to size limitations. The full set of enriched terms, including full names, and associated genes is presented in Additional file 5. Enrichment score equals Log(1/corrected P-value)

Similarities in the gene regulation at 1 and 6 h post infection was also observed when GO-term enrichment analyses were performed for the up-regulated genes (Fig. 4, Additional file 5). At both 1 and 6 h post infection, an enrichment of genes involved in ubiquitin-dependent protein catabolic processes were detected, which is in line with previous studies characterizing *D. discoideum* transcriptional response using microarray [22, 23]. Also in line with previous studies in *D. discoideum*, we detected an up-regulation of tRNA-synthetases at 6 h post infection [22, 23]. In addition to tRNA-synthetases, a wide range of genes predicted to be involved in several aspects of tRNA metabolism, e.g. tRNA splicing and modification, were also up-regulated mainly 6 h post infection, but also 1 h post infection (Additional file 3, Additional file 5). Furthermore, *L. pneumophila* infection appears to induce the production of reactive oxygen species (ROS) in *D. discoideum*. For both time points there was an enrichment for the GO-term L-ascorbic acid binding. In human immune cells, ROS are produced in order to kill off any invading pathogen. This process, known as the oxidative burst, leads to the accumulation of L-ascorbic acid within the cell, which is thought to protect the host from oxidative damage [40]. The ROS production in infected *D. discoideum* cells is further corroborated by up-regulation of genes for the Toll-Interleukin (TIR) receptor domain-containing protein and NADPH oxidase, previously shown to be required for ROS production, as well as a gene for superoxide dismutase [23, 41]. Altogether, the up-regulation of genes involved in both ROS production and scavenging, indicates that *D. discoideum* induce ROS production in response to *L. pneumophila* infection.

### Reduced ribosome biogenesis and energy production in *L. pneumophila* infected cells

Similar to previous reports, a down-regulation of many ribosomal protein genes were detected at 1 and 6 h post infection (Additional file 3) [22, 23]. However, our data also indicate a more global inhibitory effect on the translational machinery in *D. discoideum* after *L. pneumophila* infection. Ribosome biogenesis factors such as PeBoW and Noc complex genes are down-regulated already 1 h post infection. Both of these complexes are required for ribosome maturation [42, 43]. Also, *L. pneumophila* infection appears to affect ribosomal RNA transcription as demonstrated by down-regulation of the RNA polymerase I complex. However, the levels of rRNA could not be determined using the RNA-seq data due to poly(A) selection of the RNA. The inhibition of genes associated with snoRNA binding and function and rRNA primary transcript binding indicates that also rRNA processing and maturation are impaired in infected cells. Taken together, this indicates that *L. pneumophila* actively starts to inhibit the translational machinery in *D. discoideum* almost immediately after uptake.

*L. pneumophila* infection also caused inhibition of genes associated with primary energy metabolism pathways (e.g. GO terms pdh complex and mitochondrion in Fig. 4). However, in contrast to the effect on the translational machinery, down-regulation of energy metabolism was not detected until 6 h after infection (Fig. 4). Inhibition of genes coding for pyruvate dehydrogenase complex proteins, as well as genes for ATP citrate synthase and acetyl-CoA carboxylase A, indicates an impairment in acetyl-CoA metabolism affecting both energy production via the citric acid cycle and synthesis of fatty acids. In addition, the down-regulation of many genes associated with the mitochondrial electron transfer chain further support that energy metabolism is reduced in *L. pneumophila* infected cells.

### Common transcriptional responses to *M. marinum* and *L. pneumophila* infection

The characterization of the differentially regulated genes in *D. discoideum* after infection with *M. marinum* and *L. pneumophila,* respectively, indicated that the two pathogens induce very different responses. This is not surprising since *L. pneumophila* and *M. marinum* follow different routes within the cell after uptake, a fact that makes it difficult to set a defined time point where both pathogens have reached the same stage of infection.

**Fig. 5:**
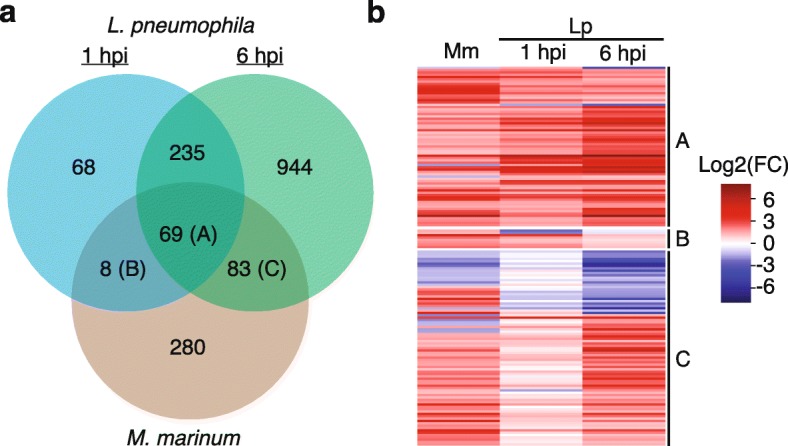
Overlap of transcriptional response during *M. marinum* and *L. pneumophila* infection. **a** Venn diagram displaying the number of *D. discoideum* regulated genes that overlap between the responses to *M. marinum* and *L. pneumophila* (1 hpi and 6 hpi) infections. **b** Hierarchically-clustered heat maps showing the regulation of each gene in the overlaps (A, B, and C) presented in the Venn diagram **a**, where Mm and Lp denote *D. discoideum* infected with *M. marinum* and *L. pneumophila*, respectively

In order to get a better resolution of the pathogen induced expression, we compared the profiles of differentially expressed genes for each bacterium. Indeed, the majority of the regulation is specific for each pathogen and there was a greater overlap of regulated genes between the two time points of *L. pneumophila* infected *D. discoideum* cells than when cells infected by *L. pneumophila* were compared to *M. marinum* infected cells (Fig. 5a). However, a substantial overlap of 160 genes, that were differentially expressed in response to both *M. marinum* and *L. pneumophila* was also identified (Fig. 5a, section A, B and C). Interestingly, the majority of these genes show similar regulation in response to both pathogens (Fig. 5b). Among these, there are several small GTPases and iron metal transporters, e.g. *nramp1* (Additional file 6). Notably, there is also an induction of several RNA interferences (RNAi) machinery genes in response to both pathogens.

The common transcriptional response in *D. discoideum* also covers genes which previously have been associated with only one of the pathogens. For example, ten of these genes are annotated as either **I**nduced or **R**epressed after **L**egionella **I**nfection (ili/rli) [25]. However, our data demonstrate that nine out of ten of these genes are regulated in similar ways upon infection by either pathogen (Additional file 6). Also one of the three vacuolin genes, *vacA*, was up-regulated in response to both pathogens. The vacuolins in *D. discoideum* are similar to mammalian late endosome associated flotillins [44] and depletion of *vacB* has been demonstrated to cause decreased proliferation of *M. marinum* in *D. discoideum* cells [12]. Taken together, the comparison of the differentially expressed genes identified after infection by *L. pneumophila* and *M. marinum* shows that the two pathogens trigger distinct transcriptional responses. However, there is also a substantial overlap including the RNAi machinery, which might be part of a general, rather than pathogen specific, response to infection.

### The response to intracellular infection is distinct from the response triggered by food bacteria

In nature, *D. discoideum* feeds on bacteria and other microorganisms, which are taken up by phagocytosis and subsequently degraded to supply the amoeba with nutrients [1]. Hence, we considered that the common gene expression response, induced by pathogenic *M. marinum* and *L. pneumophila*, may be part of a general process used for uptake of any bacteria. To investigate this, the regulated genes in *M. marinum* and *L. pneumophila* infected cells were compared to the transcriptional changes 2 h after the addition of *Escherichia coli B/r* previously studied by microarray transcriptome profiling (cluster 1, 4, 5 and 7 in [45]) (Additional file 6). *E. coli B/r* is considered non-pathogenic and is commonly used as food source for *D. discoideum* in the laboratory [25]. Since the microarray design covered only ~ 70% of the genes in *D. discoideum*, we limited the comparison to the genes represented on the microarray.

**Fig. 6:**
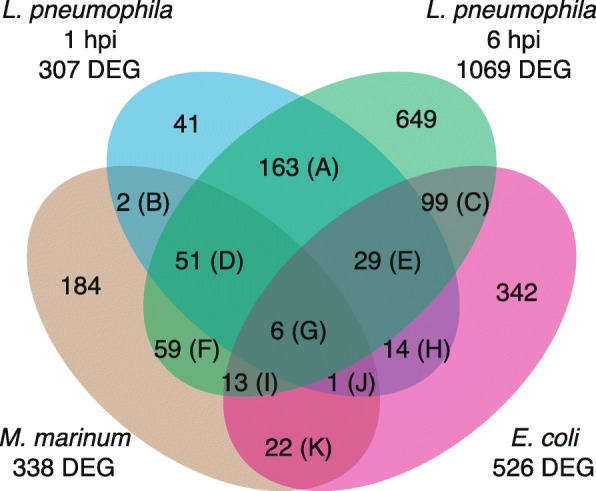
Comparison of transcriptional response to pathogenic and food bacteria. Numbers represent differentially expressed genes (DEG) of *D. discoideum* in response to *L. pneumophila* and *M. marinum* as determined by RNA-seq and *E. coli* as previously analyzed with microarray [45]. Only genes represented on the microarray chip were included in the comparison. Colored ellipses represent different bacterial challenges. (A)-(K) denotes DEG that are common to two or more bacterial challenges/time-points and hpi indicates hours after infection

The fractions of regulated genes unique and common to the two *L. pneumophila* infections and the *M. marinum* infection were almost identical when the genes not represented on the microarray were excluded from the comparison (Figs. 5 and 6), validating the approach to compare RNA-seq data and the microarray analysis. As for *L. pneumophila* and *M. marinum* infection, most of the genes (~ 65%) that were differentially regulated upon challenge with the food bacteria *E. coli* were unique, i.e. not affected by infection with the pathogenic bacteria. However, a common set of 162 genes were differentially regulated in response to *L. pneumophila* (one and six hpi) and *E. coli* (Fig. 6: C, E, G, H, I and J). Also, 42 differentially expressed genes were found to overlap between the *M. marinum* and *E. coli* challenge (Fig. 6: G, I, J and K) while only 20 regulated genes were shared between the responses to all three bacteria (Fig. 6: G, I and J).

Although there are overlaps between genes differentially regulated when *D. discoideum* is challenged with *E. coli B/r* and either of the two pathogens, closer inspection reveals large discrepancies in the responses to pathogenic and food bacteria. Similar to what we previously described (see above), the great majority of the genes, 82–84%, affected in response to both *M. marinum* and *L. pneumophila* show similar regulation also when only genes included on the microarray are included in the comparison (Additional file 1: Figure S5). In contrast, only 24–50% of the genes show similar regulation in response to *E. coli* and either of the pathogens (Additional file 1: Figure S5, Additional file 6). Of the 20 genes that were differentially expressed in response to all three bacteria, nine had common regulatory response where six were up- and three were down-regulated (Additional file 6). In summary, comparison of transcriptional regulation revealed that the responses triggered by the pathogenic bacteria *M. marinum* and *L. pneumophila* are overall distinct from that of the food bacteria *E. coli B/r*.

### *D. discoideum* transcriptional response to pathogens is evolutionary conserved

**Fig. 7:**
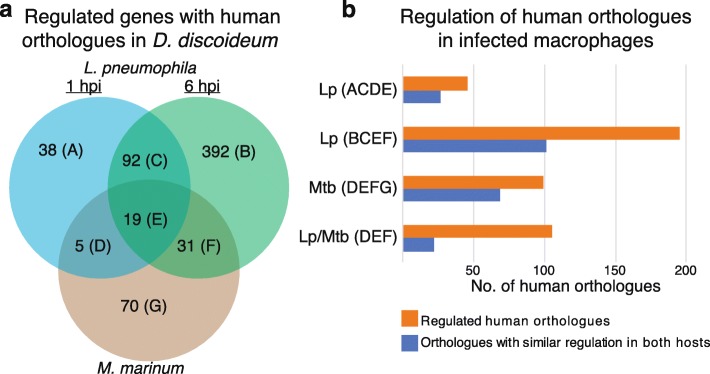
Identified orthologues and their regulation in response to infection of *D. discoideum* and human macrophages. **a** Number of differentially regulated *D. discoideum* genes identified as orthologues to human genes. **b** Number of regulated human genes orthologous to genes in *D. discoideum* (denoted as *capital letters* corresponding to letters in the Venn diagram in **a**) in response to *L. pneumophila* (Lp) [20] or *M. tuberculosis* (Mtb) [19]. In the comparison with both Lp and Mtb (Lp/Mtb), similar regulation refers to the similar differential expression in response to either Mtb, Lp or both. Full list of identified orthologues and their regulation in *D. discoideum* and macrophages is available in Additional file 7

Next, we investigated if the transcriptional responses in *D. discoideum* triggered by infection with *M. marinum* and *L. pneumophila* are conserved, i.e. if similar responses can be detected in infected human cells. We first searched for human orthologues to the ~ 12,200 protein coding genes in *D. discoideum*, resulting in 4649 *D. discoideum* genes that were orthologous to 6123 genes in the human genome (see Materials and methods for detailed information). Next, we cross-examined these orthologues with the list of all genes differentially regulated in *D. discoideum* when challenged with *L. pneumophila* and *M. marinum* (see above) (Additional file 7). This analysis revealed human orthologues for ~ 30% (125 out of 440) of the genes differentially expressed in *D. discoideum* when infected with *M. marinum* (Fig. 7a). For *D. discoideum* infected with *L. pneumophila*, human orthologues were found for ~ 40% of the differentially regulated genes at both 1 hpi and 6 hpi, i.e. 154 out of 380 genes and 534 out of 1331 genes, respectively (Fig. 7a). Finally, we investigated if these human orthologues also were differentially expressed in human cells when challenged with pathogens. For this we analyzed available data sets for transcriptional responses in human monocyte derived macrophages (HMDM) infected with *M. tuberculosis* [19] and *L. pneumophila* [20]. In total, more than 500 of the human genes orthologous to differentially regulated *D. discoideum* genes were regulated also in macrophages infected with *M. tuberculosis*, *L. pneumophila* or both bacteria (Additional file 7). The majority of these orthologues show similar expression pattern for each pathogen upon infection in both *D. discoideum* and macrophages (Fig. 7b). This trend is less pronounced for the human orthologues to the common set of 55 *D. discoideum* genes (Fig. 7a: D, E, F) that are differentially expressed in response to both *M. marinum* and *L. pneumophila* in *D. discoideum* (Fig. 7b). Taken together, human orthologues were identified for approximately 40% of the genes involved in host response to mycobacteria and *L. pneumophila* infection in *D. discoideum* and in most cases they showed similar regulation in both hosts.

### Conserved genes differentially expressed in *D. discoideum* and human macrophages upon infection

**Fig. 8:**
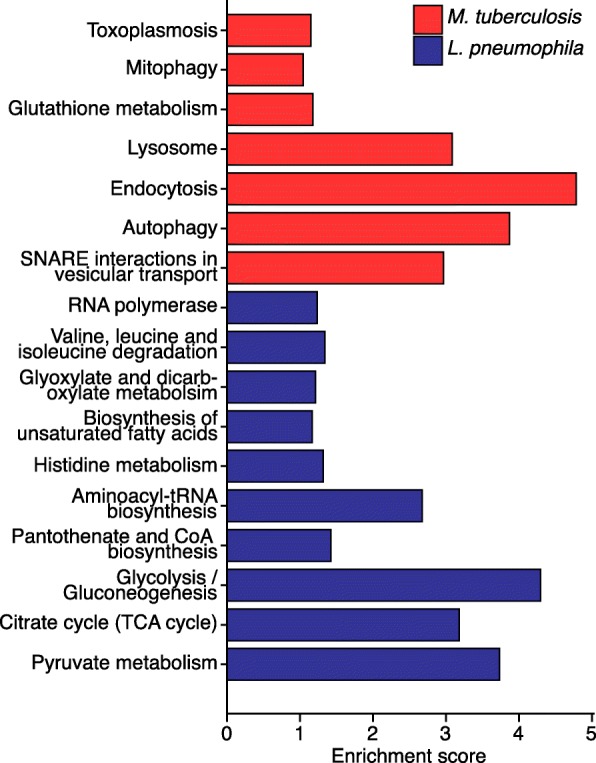
KEGG pathway analyses of regulated human orthologues. Enriched pathways (adjusted P-value < 0.1) for human-*D. discoideum* orthologous genes regulated in human monocyte derived macrophages (HMDM’s) in response to either *M. tuberculosis* or *L. pneumophila*

KEGG pathway analyses based on the differentially regulated human orthologues showed several enriched pathways such as “Endocytosis” in response to mycobacteria and “Pyruvate metabolism” in response to *L. pneumophila* (Fig. 8, Additional file 7). The majority of these pathways are also related to the enriched GO terms we identified in *D. discoideum* in response to *M. marinum* and *L. pneumophila* infections (Figs. 3 and 4). In addition, more similarities were found when we manually inspected the orthologues regulated in both hosts. Taken together, we identified an up-regulation of small GTPases, e.g. RRAS2, RAB8B, RAB13, and ARHGAP24, which regulates actin rearrangement, in HMDM’s in response to mycobacteria infection similar to the regulation of small GTPases in *D. discoideum*. Also, many genes involved in autophagy were up-regulated in macrophages e.g. GABARAPL1–3 and WIPI1 as well as several E3 ubiquitin-protein ligases. Finally, an induction was observed for ESCRT-I and ESCRT-III gene VPS37A and CHMP5, as well as other ESCRT associated genes, e.g. PDCD6IP and IST1.

Even though less similarities were found among the down-regulated orthologues in response to mycobacteria in *D. discoideum* and macrophages, some orthologues such as glutathione S-transferases, involved in detoxification of xenobiotic substances [46], showed similar regulation in both hosts.

In *D. discoideum, L. pneumophila* infection induce an up-regulation of E3 ubiquitin-protein ligases genes, tRNA synthetase genes and genes involved in ROS production and scavenging (see above). Of these, only E3 ubiquitin-protein ligases, e.g. ARIH1 and TRAF6, are represented among the orthologues with similar regulation in response to *L. pneumophila* infection in both *D. discoideum* and macrophages. However, a clear repression of genes associated with ribosome biogenesis was detected in both hosts (Additional file 7). As in *D. discoideum*, *L. pneumophila* infection triggers a down-regulation of both cytoplasmic and mitochondrial ribosomal protein genes, e.g. RPS27L and MRPL52, as well as genes associated with ribosome assembly, e.g. RRS1 and NOP10. In addition, DNA-directed RNA-polymerase I components POLR2H and ZNRD1 are down-regulated in both hosts, indicating a decrease in ribosomal RNA transcription in response to *L. pneumophila* infection. Also the down-regulation of e.g. DDX27 and PeBoW complex genes (PES1 and WDR12) indicates that the maturation of ribosomal RNA is impaired in both hosts. In summary, the regulation of many key genes*,* e.g. autophagy in response to mycobacteria and ribosome biogenesis in response to *L. pneumophila*, was recapitulated in human macrophages, supporting the relevance of *D. discoideum* as an infection model.

## Discussion

Over the past decades, *D. discoideum* has emerged as a powerful model system to investigate interaction between intracellular pathogens and their host (reviewed in [1–4]). In particular, *D. discoideum* is commonly used as host model to study the infection routes of *L. pneumophila* and *M. marinum*, a model for *M. tuberculosis*. Besides detailed studies of the impact of individual genes and proteins during the infectious process, microarray transcriptional profiling have been used to investigate the global host response to *L. pneumophila* [22, 23] and *Salmonella typhimurium* [47]. In this study, we report high-throughput RNA-sequencing to characterize the transcriptional response in *D. discoideum* upon infection by *M. marinum* and *L. pneumophila.* The analyses were performed at early time points after infection in order to identify genes that are regulated in *D. discoideum* when the pathogen actively manipulates the host to ensure survival.

### Transcriptional signature of *M. marinum* infection in D. discoideum

The majority of the genes found to be affected by *M. marinum* infection show increased expression. Many of these genes are involved in actin dynamics, such as genes encoding actin, acting regulating small GTPases, and actin binding proteins. This effect on actin related processes is not surprising since the actin cytoskeleton, and its regulation by GTPases, are involved in a wide range of cellular processes including phagocytosis, intracellular trafficking and autophagy and is therefore often a target for manipulation by pathogens [48]. One of the up-regulated genes, *comA*, encodes the actin binding protein comitin, which has been shown to associate with early phagosomes in *D. discoideum*. Cells lacking comitin are impaired in phagocytosis of *E. coli* and particles of *S. cerevisiae* [49], indicating a general role in phagocytosis. However, our study shows that transcripts of *comA* only increase in response to *M. marinum* and not *L. pneumophila* or *E. coli B/r* suggesting a role for comitin also after uptake, at least for *M. marinum* infection. Actin also plays an important role when *M. marinum* infects *D. discoideum* [3]*,* which is reflected in our RNA-seq analyses. However, our transcriptome analyses do not exactly mirror all previously reported steps of actin dynamics during infection. Previous reports have demonstrated that actin accumulate at the MCV early after uptake [9, 12], a process later shown to be dependent on the Wiskott-Aldrich syndrome protein and the SCAR Homolog (WASH) complex [50]. Also, cells lacking the actin binding protein coronin A, encoded by *corA*, or the actin polymerization regulating GTPase *racH* are more permissive for *M. marinum* proliferation in later stages of infection [12, 51]. However, we detected no difference in the levels of transcripts from *racH, corA* or WASH associated genes upon *M. marinum* infection, indicating that the regulation of these components is not triggered by different RNA levels but more likely relies on differential protein expression and/or protein re-localization.

In addition to the actin related transcriptional response, Rab family GTPases were also up-regulated upon *M. marinum* infection. In human cells, this family of proteins takes part in the regulation of intracellular vesicular transport [28] and many are actively manipulated in macrophages upon *M. tuberculosis* infection in order to alter the endocytic pathway (reviewed in [52]).

Two other transcriptional hallmarks of mycobacteria infection found in *D. discoideum* were the induction of genes associated with the autophagic and ESCRT machineries. ESCRT proteins are required for autophagy and have traditionally been associated with cytokinesis, budding of HIV-1, and multivesicular bodies (MVB) biogenesis. However, recently ESCRT and its associated proteins have been implicated in a wide range of different biological processes (reviewed in [35]). Notably, *M. tuberculosis* interferes with the ESCRT machinery in macrophages which in turn prevents normal phagosome maturation [31, 32]. The ESCRT related response in *D. discoideum* infected with *M. marinum* includes up-regulation of the majority of the genes coding for ESCRT-I and ESCRT-III components. In addition, several genes coding for ESCRT associated proteins e.g. Vps4 and Vta1, were also induced. Hence, our results indicate a substantial involvement of the ESCRT machinery when *D. discoideum* is infected with *M. marinum*. These findings are further strengthened by a recent study which showed a recruitment of ESCRT-1 component Tsg101, ESCRT-III component Vps32, and Vps4 to the MCV already 1.5 hpi in response to *M. marinum* infection in *D. discoideum* [53]*.*

Traditionally, autophagy denotes a cellular process connected to the degradation and recycling of cellular components. More recently, autophagy has been assigned a wide range of additional functions including defense response to pathogenic bacteria, often referred to as xenophagy [39]. Autophagy has been shown to be involved in the response to mycobacteria, and can also be manipulated by the bacteria, both in *D. discoideum* and human cells [9, 12, 15, 36–38]. This is also reflected in our data, where most autophagy genes previously identified to be part of the response to *M. marinum* infection in *D. discoideum* are up-regulated. In addition, we also observed induction of genes encoding the Atg5-Atg12 complex, which localizes to the phagophore membrane, and the gene for the proposed autophagy receptor CnrD [39]. The role of the host cell’s autophagic machinery during mycobacteria infection highlights the complex dynamics of host-pathogen interactions. In *D. discoideum*, autophagy is involved in restricting the growth of *M. marinum*, as demonstrated by reduction in bacterial load after induction of autophagy. At the same time, the pathogen uses the autophagic machinery in order to establish normal MCVs where the bacteria can survive and proliferate [15].

### Transcriptional signature of *L. pneumophila* infection in D. discoideum

Early host response to *L. pneumophila* infection has previously been investigated in *D. discoideum* by microarray transcriptome analysis [22, 23]. However, by using high-throughput RNA-seq, we acquired a more extensive picture of the transcriptional response. There are several advantages with RNA-seq compared to microarray, e.g. broader dynamic range [27] and the ability to analyze the effect on all genes, not limited to those represented on the microarray chip. In addition, since the same batch of RNA was used for RNA-seq and the 6 hpi microarray analyses [23], we could compare the two methods.

Despite the fact that we used a quite conservative cut off (FDR < 0.05), RNA-seq identified many more genes as differentially regulated compared to the microarray studies. Previously, only 19 differentially expressed genes had been identified at 1 h post infection [22], compared to the 380 genes identified by the RNA-seq analysis. The same trend was observed 6 h post infection, where RNA-seq identified ~ 1330 differentially expressed genes as compared to the ~ 900 recognized by microarray [23]. In general, the genes identified with RNA-seq but not microarray are involved in similar biological processes as previously described but contribute to a more detailed picture of the transcriptional response to *L. pneumophila* infection. It should be noted that the larger number of affected genes identified by RNA-seq was not only due to the limited number of genes represented on the microarray chip.

Our analyses indicated a large increase (three-fold) in the number of differentially regulated genes at 6 h post infection as compared to 1 h after *L. pneumophila* infection. Interestingly, 95% of the differentially expressed genes at 6 h post infection were also similarly affected, albeit with much weaker regulation, already at 1 h post infection. This indicates that the transcriptional reprogramming after *L. pneumophila* infection is established very early after uptake and maintained at least until 6 h post infection. This is in agreement with findings that *L. pneumophila* can manipulate the host, probably via the translocation of effector proteins, within minutes after uptake [54].

One of the most dramatic transcriptional response to *L. pneumophila* infection in *D. discoideum* was the down-regulation of genes associated with the translation machinery. Even though our RNA-seq data suggest a major inhibitory effect of translation, the effect could be even more pronounced since *L. pneumophila* may also down-regulate translation directly. In macrophages, *L. pneumophila* induce translational inhibition, via translocation of effector proteins, which is counteracted by the host through selective translation of proteins required for inflammation [55]. Effector proteins from the pathogen also block the unfolded protein response (UPR) of the host, which is triggered by misfolded proteins and endoplasmic-reticulum (ER) stress, and thereby prevents an induction of innate immune response signaling [56, 57]. This interaction between the host and the pathogen appears to be conserved as *L. pneumophila* defective in UPR-inhibition show defective growth in *D. discoideum* [58]*.* In addition, *L. pneumophila* has been shown to manipulate the cell cycle in macrophages by inhibiting translation via the translocation of protein synthesis inhibitors [59]. Control of the host’s cell cycle is important for the pathogen as the intracellular replication of *L. pneumophila* is inhibited when the host cell is in the S-phase [60].

In addition to reduced mRNA levels of genes involved in translation machinery, we also observed a major reduction of expression of genes involved in ATP production via respiration. This down-regulation is analogous to the effect on energy metabolism in macrophages, where *L. pneumophila* manipulates the mitochondria to reduce respiration, which in turn promotes proliferation of the bacteria [61]. Reduced energy production in *D. discoideum* cells is further supported by the down-regulation of mitochondria-encoded genes involved in energy production (*nad5*, *atp6*, *cytB* and *cox3*) upon *L. pneumophila* infection [62]. The regulation of these genes is not available in our data due to the poly(A) enrichment of the RNA samples prior to sequencing.

### Intracellular pathogens and food bacteria induce different transcriptional responses

The GO-term enrichment analyses of genes regulated after infection by *M. marinum* and *L. pneumophila* indicated that the two pathogens induce very different transcriptional responses in *D. discoideum*. Indeed, a comparison revealed that the majority of the regulated genes were unique for the response to each pathogen. However, we also detected 160 genes regulated in response to both pathogens of which the majority also showed similar regulation. Many of these common genes are not well characterized, but the overlap may represent a common defense response to pathogenic bacteria in *D. discoideum.* To corroborate this, we compared our results with previously published microarray data from *D. discoideum* cells grown on *E. coli*. The result strongly indicated that the common gene response elicited by the pathogenic bacteria is different from the genes affected by food bacteria.

### Transcriptional response in *D. discoideum* is conserved in human macrophages

The transcriptome analyses of *D. discoideum* infected with *L. pneumophila* and *M. marinum* revealed distinct changes in gene expression and identified cellular pathways modified by the pathogens. Are the same genes and processes affected in human cells upon challenge by pathogens? To answer this, we searched for human orthologues to the genes differentially regulated in *D. discoideum* and investigated if these were differentially expressed also in macrophages infected with *L. pneumophila* or *M. tuberculosis* [19, 20]. About 40% of the differentially regulated genes *D. discoideum* have human orthologues and the majority of the regulated human orthologues were differentially expressed in the same manner in both hosts. These includes genes for GTPases, autophagy, and ESCRT (mycobacterial infection) and genes for tRNA synthetases, ROS production and ribosome biogenesis (*L. pneumophila* infection). Although we identified many genes with similar functions and regulation in both *D. discoideum* and human macrophages, this is most likely an underestimation since the search for orthologues were based solely on amino acid sequence. Due to the evolutionary distance between the amoebae and human, further information is needed to identify additional orthologues. For example, the human Argonaut protein genes, AGO2, AGO3, and AGO4, are up-regulated in macrophages in response to *M. tuberculosis* [19]*.* These proteins have similar domain composition as the *D. discoideum* Argonaute-like protein AgnB, which is up-regulated in response to *M. marinum*, but are not identified as orthologues based on amino acid sequences alone. In recent years, a large number of studies has shown that host miRNAs are key players in regulating immunity pathways in mammalian cells and that several bacterial pathogens, including *M. tuberculosis*, can manipulate the host miRNA expression in order to avoid degradation (reviewed in [63]). Perhaps the same applies to the miRNAs identified in *D. discoideum* [64–67].

## Conclusions

The amoeba *D. discoideum* has for many years been used as a model to study the interplay between the host and the pathogen during infection. These studies have mainly dealt with detailed investigations of how specific genes and proteins affect the infection process. In this report we take a global approach, using high-throughput RNA sequencing to analyze the effect on gene transcription upon infection by *M. marinum* and *L. pneumophila*. Both pathogens induce a strong transcriptional response of the host early after uptake. The transcriptional signatures identified correspond well with previous published studies, but also add valuable new knowledge concerning the complex interaction between host and pathogen. In addition, the transcriptional response in *D. discoideum* is in many aspects similar to the response observed for infected macrophages, emphasizing the relevance of *D. discoideum* as a model for infection.

## Materials & methods

### *Dictyostelium discoideum* cell culture

*D. discoideum* AX2–214 (DBS0235534, www.dictybase.org) was used for all experiments in connection with *M. marinum* infection and was kindly provided by Thierry Soldati. *D. discoideum* cells were cultured axenically at 22 °C in 10 ml HL5-C medium (pH 6.4) (Formedium) supplemented after autoclaving with 1% glucose and 100 U/ml Penicillin-Streptomycin (Gibco by Life Technologies). The cells were grown in BD Falcon tissue culture dishes (100 × 20 mm). Antibiotics were excluded from the growth medium the day before and during infection experiments.

### Mycobacterial strains and growth conditions

*Mycobacterium marinum* M strain carrying the msp12::*gfp* plasmid, constitutively expressing GFP, was kindly provided by Thierry Soldati and used in the infection experiments [12]. Bacteria were grown on 7H10 agar (Difco Middlebrook, BD) containing 10% OADC supplement (Difco Middlebrook, BD) and 0.5% glycerol, or cultivated in 7H9 broth (Difco Middlebrook, BD) supplemented with OADC, 0.2% glycerol and 0.05% Tween80 (Sigma Aldrich) at 32 °C shaking culture with glass beads to decrease bacterial clumping. Both 7H10 agar and 7H9 broth were supplemented with 50 μg/ml kanamycin to maintain the msp12::*gfp* plasmid.

### *M. marinum* infection and cytotoxicity assays

The day before infection, BD Falcon tissue culture dishes (100 × 20 mm) were seeded with *D. discoideum* AX2 cells and grown without antibiotics overnight to 2 × 10^7^ cells per plate. *M. marinum* were collected and resuspended in HL5-C, bacterial clumps were disrupted by passing through a blunt end 26-gauge needle after which bacteria were added to the amoeba at MOI ~ 200 unless otherwise specified. Non-infected samples were prepared simultaneously and treated identically except for the addition of bacteria. The infection was initiated by centrifugation at 500×g twice for 7 min or three times for 5 min followed by incubation at 25 °C for 50 min post infection to allow for uptake of bacteria. Subsequently, extracellular *M. marinum* cells were removed by five washes with HL5-C after which the cells were incubated at 25 °C. The cells were collected in HL5-C approximately 2 h after initiation of infection and a subsample was added to the same volume of Soerensen buffer (2 g KH_2_PO_4_ and 0.27 g Na_2_HPO_4_ in 1 L water, pH 6) + 5 mM Sodium azide (Sigma). Subsamples from infections for RNA-seq were analyzed in a BD Accuri C6 flow cytometer in Soerensen buffer + 20 mM sorbitol. Subsamples for independent RT-qPCR and cytotoxicity tests were analyzed on MACSQuant® VYB Flow Cytometer (Miltenyi). To determine viability of *D. discoideum* cells after challenge with *M. marinum*, infections were performed as described above, with MOI 0, 1, 10, 100 and 200. The fraction of dead (permeable) cells were analyzed by staining the cells with 4 μM propidium iodide (generous gift from Mikael Sellin) in Soerensen buffer + 20 mM sorbitol for 15 min prior to flow cytometric analysis [21]. Cell viability was determined by gating as shown in Additional file 1: Figure S1a.

### High-throughput sequencing

Two separate *M. marinum* infection experiments were prepared for RNA-seq. Total RNA was isolated by the Ambion PureLink™ RNA Mini kit in combination with TRIzol (Invitrogen), followed by on column DNase treatment according to the manufacturer’s instructions (Ambion, Invitrogen). RNA yield and quality were measured by Nanodrop (Thermo Scientific) and by agarose gel electrophoresis, respectively. Preparation of *L. pneumophila* infected samples were described previously [23]. Total RNA samples, biological duplicates for each time point, were sent to SciLifeLab Stockholm for library preparation and high-throughput sequencing. mRNA was isolated from the total RNA pool using poly(A) extraction with oligo dT prior to sequencing with Illumina HiSeq 2000/2500 system.

### Read mapping and differential expression analysis

Reads were filtered by mapping to tRNA and snoRNA sequences [25] including 50 base pair flanking regions as well as mapping to the extrachromosomal rRNA palindrome [68] and the mitochondrial genome [69]. Finally, the remaining reads were mapped to *D. discoideum* AX4 genome [70], excluding the duplication of chromosome 2 (DDB0232429:3015460–3,766,689). All mapping steps were performed with TopHat v2.0.13 [71] and bowtie2 v. 2.2.3 [72] using default settings. Number of reads mapping to each gene, annotations accessed 2018-08-24 [25], were counted using featureCounts v. 1.6.2 [73]. Differential expression analysis and principal component analyses (rlog transformed counts) were performed using DESeq2 [26]. Unless otherwise stated, genes with a false discovery rate (FDR) < 0.05 in respective DESeq2 analyses were considered to be regulated, without indication of statistical significance. Gene ontology term enrichment analysis were performed using LAGO [74] for up- and down-regulated genes respectively for each sample type and time point. Terms with a *P*-value < 0.05 after Bonferroni correction were considered to be enriched.

### Comparison of transcriptional responses and orthologue identification

Transcriptional responses detected with RNA-seq were compared to previously reported *D. discoideum* transcriptional responses to *L. pneumophila* infection 6 h post infection [23] and 2 h after *E. coli* B/r addition (cluster 1, 4, 5 and 7 in [45]), assayed by microarray. Probe IDs were retrieved from the microarray design [24] and updated to current dictyBase [25] gene ID’s and only genes represented on the array were included in the comparisons. Human-*D. discoideum* orthologues were identified with OrthoFinder v2.2.7 [75]. Search was performed with all protein sequences from *D. discoideum* (accessed 2017-10-11 [25]) and the full reviewed proteomes for *H. sapiens, Mus musculus, Rattus norvegicus* and *Saccharomyces cerevisiae* (accessed 2017-10-11 [76]). The proteomes of *M. musculus, R. norvegicus* and *S. cerevisiae* was included to increase the sensitivity of the orthologue identification. Next, human orthologues were identified for the *D. discoideum* genes involved in the transcriptional response to *M. marinum* and *L. pneumophila*. The regulation of these genes where then analyzed in human monocyte derived macrophages (HMDM’s) in response to *M. tuberculosis* H37Rv and *M. tuberculosis* GC1237 infection from previously published data [19]. Only the 4 and 18 h post infection was included and the regulation for the two *M. tuberculosis* strains were combined since they were found to be highly similar. The transcriptional response of HMDM’s 8 h post infection of *L. pneumophila* AA100/130b was obtained from [20]. KEGG pathway analyses were performed using Enrichr [77, 78] for human genes that are both regulated in response to *M. tuberculosis* or *L. pneumophila* in macrophages and orthologous to genes regulated in response to infection in *D. discoideum*.

### cDNA synthesis and RT-qPCR

Total RNA from *D. discoideum* cultures, infected or non-infected with *M. marinum*, was reverse transcribed using RevertAid H-1st strand cDNA synthesis kit (Thermo Fisher Scientific) according to the manufacturer’s protocol. Oligo dT and random primers were used together to improve the efficiency of cDNA synthesis. Annealing temperature and specificity of each primer set was optimized by PCR with cDNA as template (See Additional file 1: Table S2). qPCR was performed using the StepOnePlus™ Real-Time PCR System (Applied Biosystems®) in 15 μl reactions including 7.5 μl Maxima® SYBR green/ROX qPCR master mix (Thermo Fischer Scientific), 0.33 μM gene specific primers, and 2 μl of 5x diluted cDNA. The cycling condition was 95 °C for 10 min, followed by 40 cycles of [95 °C for 10 s, primer-specific annealing temperature for 30 s, 72 °C for 30 s]. Differential expression between the infected and non-infected cells was calculated using the comparative Ct method, by first normalizing against the reference gene *catA* or *gpdA*, followed by normalization against the non-infected control.

## Supplementary information

**Additional file 1.** Supplementary figures, tables and additional results. **Figure S1.** Cytotoxic effect and fraction of infected cells at different multiplicity of infection (MOI) with *M. marinum.* **Figure S2.** Validation of RNA-seq by RT-qPCR when challenged with *M. marinum.* **Figure S3.** Regulation detected with microarray vs corresponding RNA-seq values. **Figure S4.** Comparison of transcriptional response to *L. pneumophila* infection one and six hours post infection. **Figure S5.** Regulation of overlapping genes in *D. discoideum* in response to *M. marinum*, *L. pneumophila* and *E. coli*. **Table S1.** Genes discussed in Additional results and in the connected Result section. **Table S2.** Primer sequences and annealing temperatures used for the RT-qPCR analyses. **Additional results** – Additional description of *D. discoideum* response to *M. marinum* and *L. pneumophila* including key genes and functions.

**Additional file 2.** Differentially regulated genes in *D. discoideum* 2.5 h post *M. marinum* infection. Regulation and description of all *D. discoideum* genes regulated (FDR < 0.05) 2.5 h post *M. marinum* infection.

**Additional file 3.** Differentially regulated genes in *D. discoideum* one and six hours post *L. pneumophila* infection. Regulation and description of all *D. discoideum* genes regulated (FDR < 0.05) one and six hours post *L. pneumophila* infection. The regulation previously determined by microarray for these genes are also included.

**Additional file 4.** Enriched GO terms for genes differentially expressed 2.5 h post *M. marinum* infection. All enriched GO terms including statistics and associated genes.

**Additional file 5.** Enriched GO terms for genes differentially expressed one and six hours post *L. pneumophila* infection. All enriched GO terms including statistics and associated genes.

**Additional file 6.** Comparison of *D. discoideum* genes regulated in response to pathogenic bacteria and food bacteria. Regulation and description of genes included in Fig. 6.

**Additional file 7.** Identified human orthologues for *D. discoideum* genes regulated in response to infection by mycobacteria, *L. pneumophila* or both. Gene IDs, regulation and KEGG pathway analyses for orthologous genes.

## Data Availability

RNA-seq data that support the findings of this study have been deposited in Gene Expression Omnibus (GEO) with the accession code GSE132461.

## Abbreviations

DEG: Differentially expressed genes
ER: Endoplasmic reticulum
ESCRT: Endosomal sorting complexes required for transport
FC: Fold change
FDR: False discovery rate
GO: Gene ontology
HMDM: Human monocyte derived macrophages
hpi: Hours post infection
ili: Induced after legionella infection
LCV: Legionella containing vacuole
Lp: *Legionella pneumophila*
MCV: Mycobacteria containing vacuole
MOI: Multiplicity of infection
Mtb: *Mycobacterium tuberculosis*
MVB: Multivesicular bodies
PC: Principal component
PCA: Principal component analysis
rli: Repressed after legionella infection
RNAi: RNA interference
RNA-seq: Illumina RNA high throughput sequencing
ROS: Reactive oxygen species
RT-qPCR: Reverse transcription quantitative PCR
T4SS: Type IVb translocation/secretion system
TB: Tuberculosis
TIR: Toll interleukin receptor
UPR: Unfolded protein response
WASH: Wiskott-Aldrich syndrome protein and the SCAR Homolog complex
