# Supplementary material for: Investigation of the host transcriptional response to intracellular bacterial infection using Dictyostelium discoideum as a host model
Source: BMC Genomics. 2019 Dec 10;20:961. doi: 10.1186/s12864-019-6269-x (PMC6902447; doi:10.1186/s12864-019-6269-x)
Supplement: Supplementary file 1 — Additional file 1. Supplementary figures, tables and additional results. Figure S1. Cytotoxic effect and fraction of infected cells at different multiplicity of infection (MOI) with M. marinum. Figure S2. Validation of RNA-seq by RT-qPCR when challenged with M. marinum. Figure S3. Regulation detected with microarray vs corresponding RNA-seq values. Figure S4. Comparison of transcriptional response to L. pneumophila infection one and six hours post infection. Figure S5. Regulation of overlapping genes in D. discoideum in response to M. marinum, L. pneumophila and E. coli. Table S1. Genes discussed in Additional results and in the connected Result section. Table S2. Primer sequences and annealing temperatures used for the RT-qPCR analyses. Additional results – Additional description of D. discoideum response to M. marinum and L. pneumophila including key genes and functions. [file 12864_2019_6269_MOESM1_ESM.pdf]

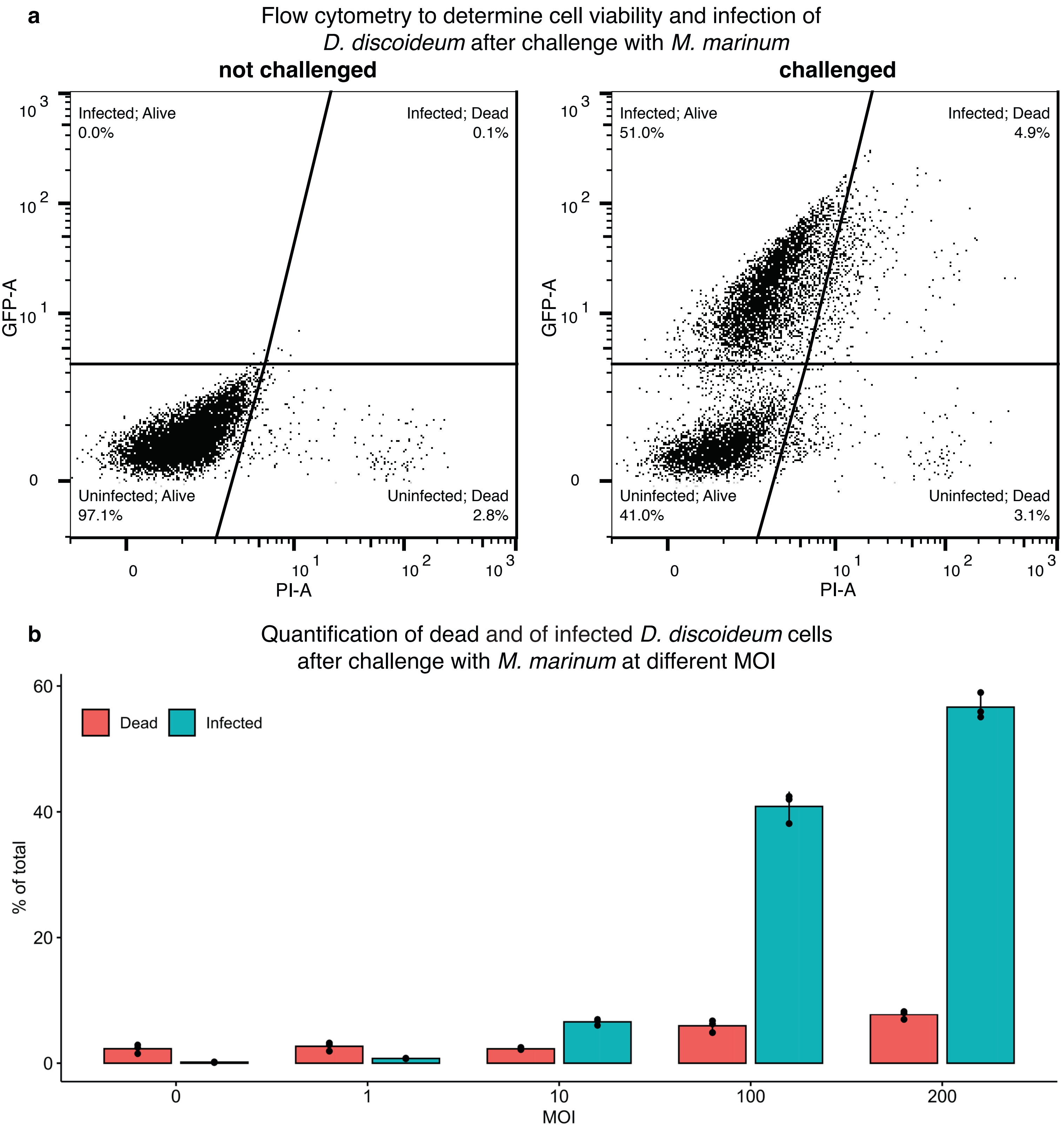

Figure S1. Cytotoxic effect and fraction of infected cells at different multiplicity of infection (MOI) with *M. marinum*. a) Flow cytomtery diagrams of propidium iodide (PI) stained *D. discoideum* cells challanged by *M. marinum* expressing GFP: not challenged (MOI = 0); challenged (MOI = 200). Populations of uninfected vs infected and alive vs dead cells were determined using gating as shown (percentage of total events indicated by numbers). b) Percentage of dead *D. discoideum* cells (red) and *D. discoideum* cells infected by *M. marinum* (blue) at indicated MOI as determined by flow cytometry. Standard error and datapoints (black points) are based on three individual infections per MOI.

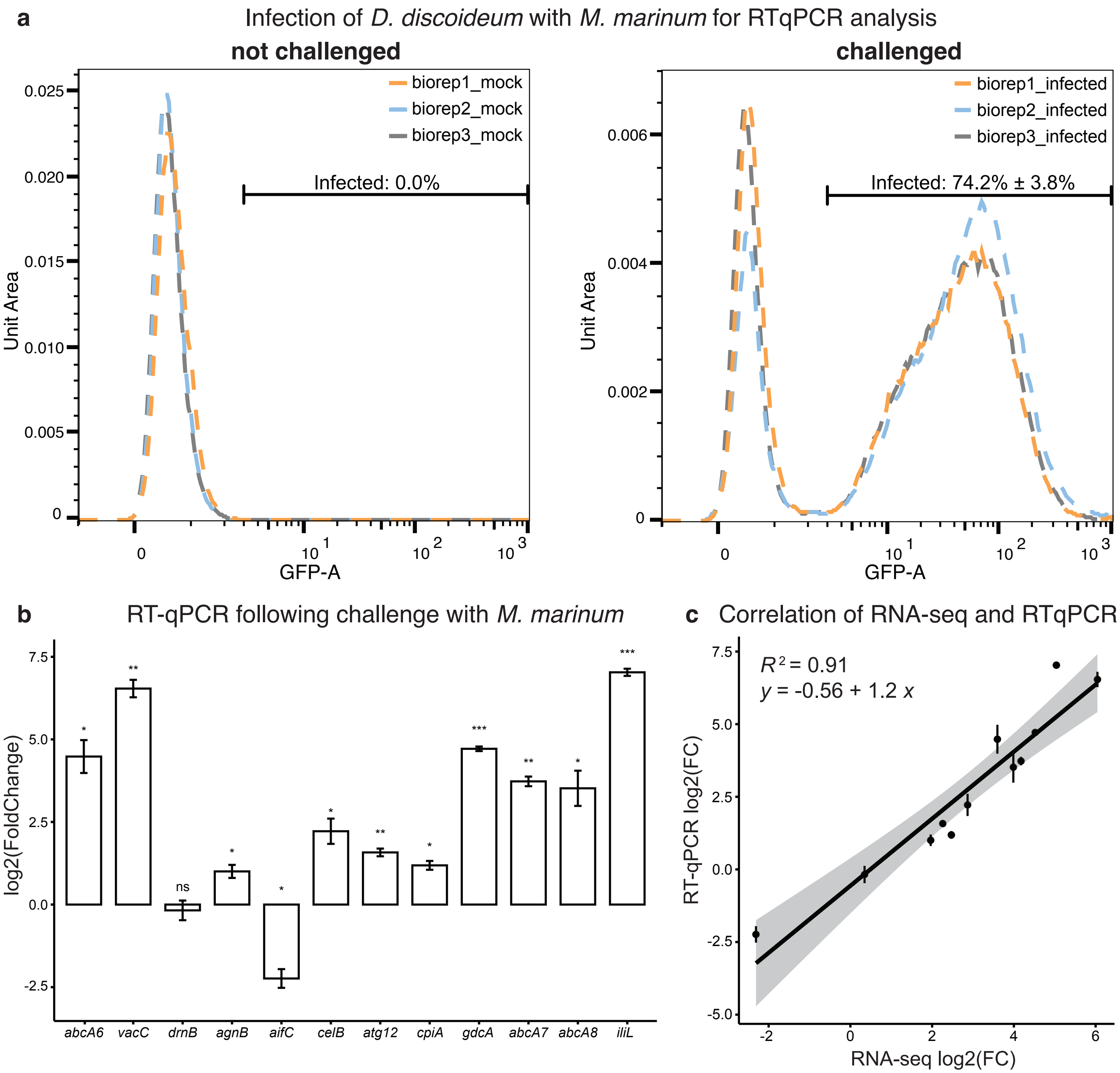

Figure S2. Validation of RNA-seq by RT-qPCR when challenged with *M. marinum*. a) Flow cytometry of three independent *D. discoideum* cultures (biorep1-3), either non-infected or infected with GFP-expressing *M. marinum* (MOI=200). Percentage of infected cells determined based on GFP intensity; numbers represent average percentage of infected cells ± standard deviation. b) RT-qPCR of RNA prepared from infections with *M. marinum* shown in (a). Average relative expression calculated by normalization to internal control and mock infection. Error bars represent standard error of the mean; statistical significance by t-test: ns = not significant, * = $p < 0.05$, ** = $p < 0.01$, *** = $p < 0.001$. (c) Scatter plot comparing log2(FoldChange) of genes in response to *M. marinum* infection as determined by RT-qPCR and RNA-seq. Pearson correlation coefficient with 95% confidence interval (gray).

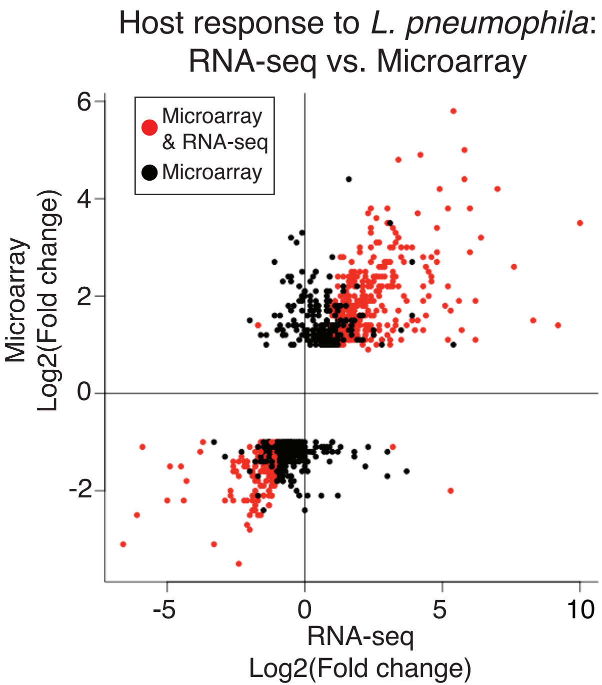

Figure S3. Regulation detected with microarray vs corresponding RNA-seq values. Comparison of gene regulation detected by microarray (p-value < 0.05 and log2(FC) > 1 or < -1) with the corresponding regulation determined with RNA-seq for *L. pneumophila* infected cells. Marked in red: significantly regulated genes according to both RNA-seq (FDR < 0.05) and microarray analyses; Marked in black: significantly regulated genes according to microarray but where the regulation detected by RNA-seq failed to meet the FDR cut off (0.05). Note that the ranges for the two axes differ.

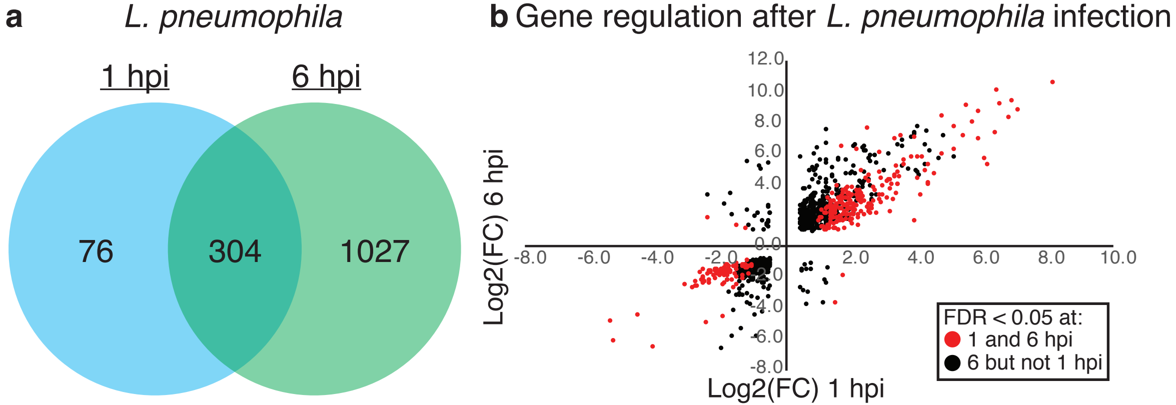

Figure S4. Comparison of transcriptional response to *L. pneumophila* infection one and six hours post infection. a) Venn diagram of the number of the regulated genes identified with RNA-seq one and six hours post *L. pneumophila* infection. b) Comparison of gene regulation at one and six hours post *L. pneumophila* infection. Marked in red: genes regulated at 1 and 6 hpi with false discovery rate (FDR) < 0.05; Marked in black: regulation of genes at 6 hpi with FDR < 0.05 and at 1 hpi using a less stringent cut off (log2(fold change) +/- 0.5).

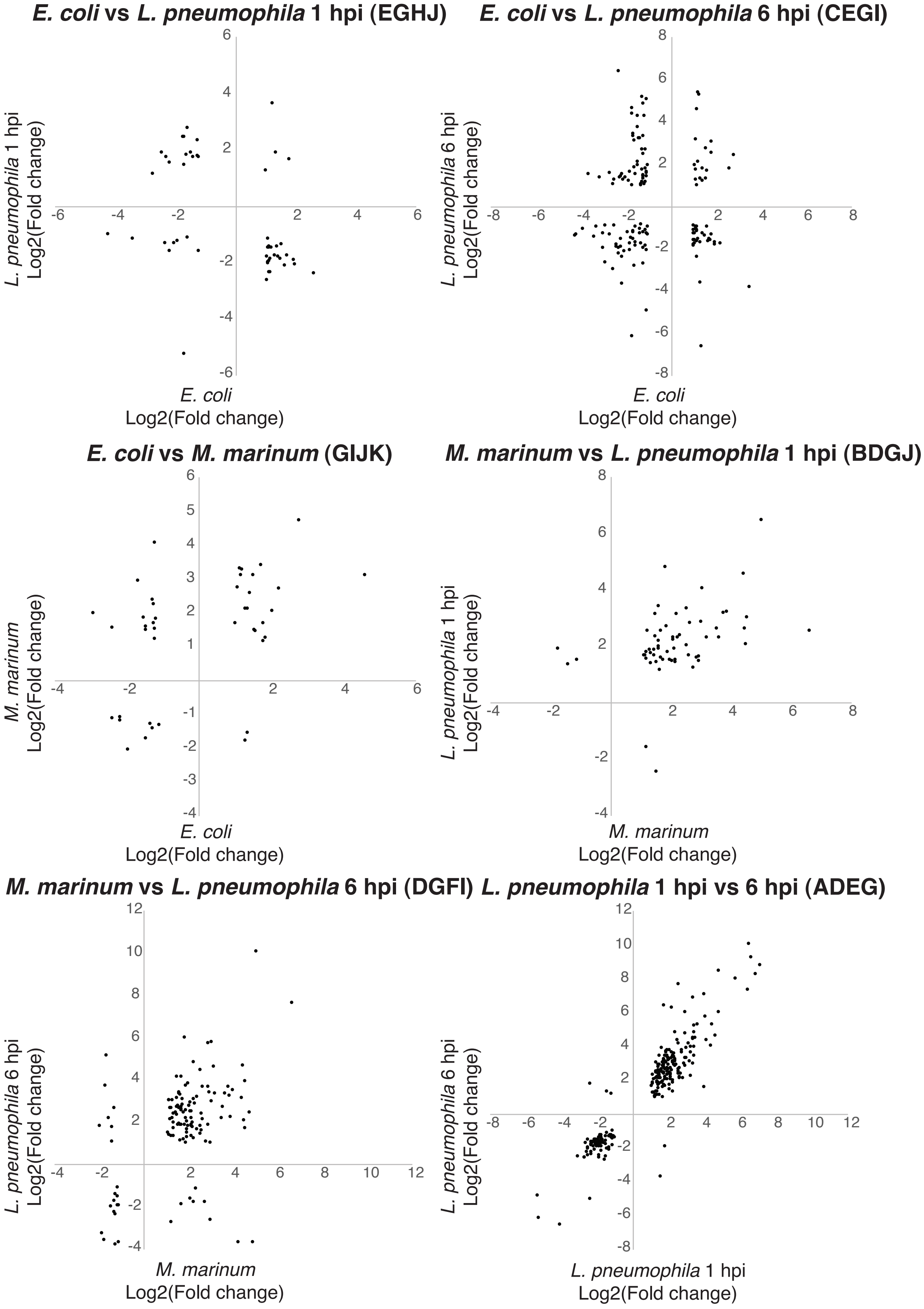

Comparison	Sections in Fig. 5	Regulation			Similar regulation
		Up	Down	Opposite	
E. coli vs *L. pneumophila* 1 hpi	E, G, H, J	4	8	38	24%
E. coli vs *L. pneumophila* 6 hpi	C, E, G,I	18	44	85	42%
E. coli vs *M. marinum*	G, I, J, K	17	4	21	50%
M. marinum vs *L. pneumophila* 1 hpi	B, D, G, J	53	17	11	82%
M. marinum vs *L. pneumophila* 6 hpi	D, G, F, I	95	13	21	84%
L. pneumophila 1 hpi vs *L. pneumophila* 6 hpi	A, D, E, G	161	59	29	88%

Figure S5. Regulation of overlapping genes in *D. discoideum* in response to *M. marinum*, *L. pneumophila* and *E. coli*. In the scatterplots, each data point represents a gene differentially expressed in response to different bacterial challenges/hpi (x- and y-axes). Capital letters (A-K) in each graph header refer back to the overlapping sections of the Venn diagram in Figure 6.

Table S1. Genes discussed in Additional results and in the connected Result section. X indicates differential expression in *D. discoideum* when infected with *M. marinum* (M.m), *L. pneumophila* (L.p.), or *E. coli* (E.c). Up and Down denotes that the genes are up- or down-regulated upon infection. Genes marked with both Up and Down are regulated in opposite directions for different bacteria. Genes not included on the microarray for the *E.coli* infection is denoted as n.i. For further information about the genes, see Additional file 6.

**Gene ID**	**Gene name**	**Description**	**M.m**	**L.p**	**E.c**	**Regulation**
DDB_G0269174	rab1c	Rab GTPase	X			Up
DDB_G0276399	rab8b	Rab GTPase	X	X		Up
DDB_G0270126	rasY	Ras GTPase	X	X		Up
DDB_G0270140	rasZ	Ras GTPase	X		n.i.	Up
DDB_G0292996	rasD	Ras GTPase	X	X	n.i.	Up
DDB_G0268622	rac1B	Rho GTPase	X	X		Up
DDB_G0277791	racO	Rho GTPase	X		n.i.	Up
DDB_G0282365	Rac1C	Rho GTPase	X			Up
DDB_G0286555	racA	Rho GTPase	X			Up
DDB_G0277849	dymA	dynamin GTPase	X		X	Up/Down
DDB_G0277851	dymB	dynamin GTPase	X			Up
DDB_G0282143	hatB	actin binding	X			Up
DDB_G0289599	comA	actin binding	X			Up
DDB_G0291081	pefA	PDCD6/ALG2 orthologue	X	X	X	Up
DDB_G0275451	alxA	PDCD6IP/Alix orthologue	X			Up
DDB_G0289149	litaf	Lipopolysaccharide induced tumor necrosis factor orthologue	X	X		Up
DDB_G0284347	vps4	ESCRT-III component	X			Up
DDB_G0271488	vta1	Similar to human VTA1 involved in endosomal protein sorting	X			Up
DDB_G0289881	atg5	autophagy	X		n.i	Up

DDB_G0282929	atg12	autophagy	X		n.i	Up
DDB_G0286191	atg8a	autophagy	X			Up
DDB_G0290491	atg8b	autophagy	X		n.i	Up
DDB_G0270098	sqstm1	autophagy receptor	X		n.i	Up
DDB_G0275209	cnrD	autophagy receptor	X	X		Up
DDB_G0292986	abcG10	ABCG family transporter	X	X		Down/Up
DDB_G0274115	abcG12	ABCG family transporter	X	X		Down
DDB_G0273073	abcG17-1	ABCG family transporter	X		X	Down
DDB_G0276973	nramp1	Orthologue to natural resistance associated to macrophages 1/iron transport	X	X	X	Down
DDB_G0269470	mcfF	Mitoferrin/iron transport	X	X		Down
DDB_G0287297	p80	Endosome/phagosome membrane protein/putative copper transporter	X			Down
DDB_G0289237	tirA	Toll-Interleukin (TIR) receptor domain-containing protein/ROS production		X		Up
DDB_G0287101	noxB	NADPH oxidase gene/ROS production		X		Up
DDB_G0283021	sodB	Superoxide dismutase/ROS production		X	X	Up/Down
DDB_G0278341		ATP citrate synthase		X	X	Down
DDB_G0288387	accA	Acetyl-CoA carboxylase A/Similar to ACLY		X	X	Down
DDB_G0284947	ucr	Ubiquinol-cytochrome c oxireductase subunit		X		Down
DDB_G0290377	agnB	Argonaute B/RNAi	X	X		Up
DDB_G0289461		CCR4-NOT complex subunit/RNAi	X	X		Up
DDB_G0284409	iliB	Induced by Legionella infection (ili)	X	X		Up
DDB_G0271068	iliC	ili/ belongs to a superfamily of metalloenzymes	X	X		Up
DDB_G0269630	iliI	ili/ TatD-related DNase , belongs to a superfamily of metalloenzymes	X	X	X	Up
DDB_G0286041	iliJ	ili/ contains a central, large T4 RNA ligase, RnlA-like domain	X	X		Up
DDB_G0278649	iliK	ili/ belongs to a superfamily of metalloenzymes	X	X		Up
DDB_G0281853	iliL	Similar to Dictystelium cell surface glycoproteins gp130, and GP138A, B, C, and D	X	X		Up
DDB_G0278481	iliN	ili	X	X		Up
DDB_G0279707	iliP	Ili/ contains a predicted signal peptide	X	X	X	Up/Up/Down

DDB_G0272783	rliA	Repressed by Legionella infection (rli)/ twelve transmembrane domain protein that may act as a transporter	X	X		Down
DDB_G0289485	vacA	Vacuolin/similar to mammalian late endosome associated flotillins	X	X		Up
DDB_G0279191	vacB	Vacuolin/similar to mammalian late endosome associated flotillins	X		n.i.	Up
DDB_G0279307	vacC	Vacuolin/similar to mammalian late endosome associated flotillins	X			Up
DDB_G0267928	tmem144b	Transmembrane transport	X	X	X	Down
DDB_G0280317		Glutathione-s-transferase	X	X	X	Down

Table S2. Primer sequences and annealing temperatures used for the RT-qPCR analyses.

Gene	Forward	Reverse	Annealing temp (°C)
agnB	TGTTTGATATCTTTGACTTGGATCA	TCCAGAATTAACATTTTTAACTGGATT	57
aifC	TGGTTTTGGAGGTAGCCAAG	TTTTTGCCAATTCTGGTTCA	57
celB	CCCTTCCACTCCAACTTCAA	CCATCGGAATTATCGGAAGA	57
atg12	TGGTGCACAACCATTAAAACA	ATTTCATCTGGGCTTGGTTG	58
abcA6	CGATTCTTTACACCTTGAATTGT	TTGTTAGAAGACAACTTTGGGATG	54
vacC	TTGATCCTCAAATTGCACTCA	TGCATCAATAGTTGCAGATGG	53
cpiA	GCCGCCAATGATGAAATTAG	CAAAACCAGCTGGGGTCTTA	59
gdcA	GGTGTTTGTGTAAAAGAAGGTGAA	GCCACAAGTTTCTCCTAAACCA	60
abcA7	CGCTATCGCTATGACTGGTG	CAGCCTCTTGCATAGAATGAGT	58
abcA8	ATTGCATCACCACTTGTTGG	ACCTTCAAAATCTGTTCATGCTT	58
iliL	TTTTAGATTCATGCGCCACA	GCTTGGGGTTGGTAAAGGTT	60
drnB	CAAGCCGATGTAATTGAAGCATTA	CGGCATCGGGTTCATCA	54
gpdA	GGTTGTCCCAATTGGTATTAATGG	CCGTGGGTTGAATCATATTTGAAC	53-60
catA	GTTTCGCTGCTCGTCAACCATACAATC	GCACGAACTTGAATTTCTTTGATGGTG	53-60

Additional results

D. discoideum response to *M. marinum* is enriched for genes involved in intracellular trafficking, autophagy and phagosome maturation.

GTP-binding proteins and actin

GTP-binding proteins commonly belong to small GTPases of the Ras superfamily. Based on sequence and functional similarities, the Ras super family members are further divided into five families: Ras, Rho, Rab, Ran and Arf (reviewed in [1]). In our data, we detected up-regulation of genes belonging to several different members of the Ras superfamily such as the Rab family e.g. *rab1c* and *rab8b*; the Ras family: *rasY*, *rasZ* and *rasD* and the Rho family: *rac1B*, *racO*, *rac1C,* and *racA* GTPases. GTPases of these families are often activated by extracellular stimuli, which in turn triggers regulation of e.g. gene expression within the cell [1]. In addition to the induction of small GTPases, the RNA-seq analysis showed that genes for dynamin GTPases, *dymA* and *dymB*, are up-regulated where DymA has been shown to associate with F-actin on the early *D. discoideum* phagosome [2]. The effect on actin dynamics was also reflected in the increased expression of e.g. *hatB* and *comA* (Additional file 2).

ESCRT and membranes

GO-term enrichment analysis showed that genes connected to Endosomal Sorting Complexes Required for Transport (ESCRT) were up-regulated in response to *M. marinum* infection. The ESCRT machinery is composed of several complexes and associated proteins and has been connected to a wide range of biological processes [3]. The three main complexes, ESCRT-I - III are conserved in *D. discoideum* [4]. Although most of the genes associated to ESCRT-I and ESCRT-III were up-regulated in response to *M. marinum* infection, ESCRT-II genes were unaffected. ESCRT-II is not essential for the function of the ESCRT machinery as ESCRT-I and ESCRT-III can be bridged via the interaction of PDCD6/ALG2, PDCD6IP/Alix and ESCRT-I component TSG101 [5]. In line with this, both the *D. discoideum* orthologues of PDCD6/ALG2, *pefA*, and PDCD6IP/Alix, *alxA,* were up-regulated in our analysis. Furthermore, we detected up-regulation of the ESCRT-associated genes lipopolysaccharide induced tumor necrosis factor (*litaf*), involved in recruitment of ESCRT-I components to cytoplasmic membranes [6], as well as Vps4-Vta1 complex genes, *vps4* and *vta1*. These findings are further strengthened by a recent study, which showed a recruitment of ESCRT-1 component Tsg101 as well as ESCRT-III components Vps32 and Vps4 to the MCV already 1.5 hpi in response to *M. marinum* infection in *D. discoideum* [7]. Furthermore, *M. tuberculosis* have been shown to interfere with the ESCRT machinery in macrophages, which in turn prevents normal phagosome maturation [8, 9].

Autophagy

Many of the genes detected as up-regulated after *M. marinum* infection are involved in different aspects of autophagy and most of these have previously been characterized mainly by gene knockouts and microscopy [10–13]. In addition, transcriptional activation of some autophagy related genes, i.e. *atg8a*, *atg8b* and *atg1*, as well as the proposed autophagy

receptor *sqstm1/p62* has been shown by RT-qPCR [13]. We detect an induction of all these genes in the RNA-seq data, except *atg1*, which failed to meet the FDR cut off. Also, our data showed increased expression of *atg5, atg12* and *atg18*. In *D. discoideum*. Atg5-Atg12 complex is associated with phagophore membrane elongation via regulation of the attachment of Atg8 to phosphatidylethanolamine (PE) in the phagophore membrane [14]. Three autophagy receptors have been proposed in *D. discoideum*; sqstm1/p62, CueA, CnrD [14]. As previously mentioned, *sqstm1/p62* is up-regulated early in *M. marinum* infection. In addition, we found that *cnrD* is up-regulated while no regulation of *cueA* was detected.

Genes for transmembrane transporters are downregulated during *M. marinum* infection.

Compared to the up-regulated genes, a smaller fraction (9%) showed reduced expression. The majority if these were enriched for GO-terms associated with transmembrane transport (Fig. 3, Additional file 4). This set of down regulated genes included genes for three ABCG family transporters, *abcG10*, *abcG12* and *abcG17* and two iron transporters, *nramp1*and *mcfF*. Surprisingly, the gene coding for the putative copper transporter *p80* [15] was down-regulated even though P80 has been shown to accumulate at the MCV [11].

***L. pneumophila* infection induces expression of genes related to reactive oxygen species ROS**

In *D. discoideum*, ROS production relies on the Toll-Interleukin (TIR) receptor domain-containing protein gene *tirA* and the NADPH oxidase genes *noxA-C* [16]. Of these genes, *tirA* and *noxB* were up-regulated both at one and six hours post infection (Additional file 3), corroborating previously reported up-regulation of *tirA* at one, four and six hours after *L. pneumophila* infection [17]. In addition, the RNA-seq analysis showed up-regulation of the superoxide dismutase gene *sodB* six hours post infection. Superoxide dismutase enzymes are involved in ROS production by converting superoxide to hydrogen peroxide [18].

Common transcriptional responses to *M. marinum* and *L. pneumophila* infection

In addition to the effect on small GTPases, iron transporters, and RNAi components, the common response to *L. pneumophila* and *M. marinum* included vacuolins, similar to flotillins, which are associated with late endosomes in mammalian cells [19]. The *D. discoideum* vacuolins are encoded by three genes, *vacA-C* [20]. One of the genes, *vacA,* was up-regulated in response to both pathogens, while *vacB* and *vacC* were up-regulated only in response to *M. marinum*. Two of them, *vacA* and *vacB,* have previously been studied during *M. marinum* infection in *D. discoideum* where depletion of *vacB* caused decreased proliferation of the pathogen while no difference was seen for cells lacking a functional *vacA* [11].

Only a minimal set of genes are commonly regulated in response to intracellular infection and food bacteria.

Altogether only 20 *D. discoideum* genes were differentially regulated in response to all three bacteria (*L. pneumophila*, *M. marinum*, and *E. coli*). Of these, nine responded in the same way, i.e. six were up-regulated and three were down-regulated. The products of all three down-regulated genes have proposed functions; two transporters, *nramp1* and *tmem144b*, and one putative glutathione-S-transferase [20]. Only two of the up-regulated genes have suggested functions, the TatD-related DNase *iliI* and the orthologue to human PDCD6/ALG2 *pefA* (Additional file 6).

References

1. Wennerberg K, Rossman KL, Der CJ. The Ras superfamily at a glance. J Cell Sci. 2005;118 Pt 5:843–6.

2. Gopaldass N, Patel D, Kratzke R, Dieckmann R, Hausherr S, Hagedorn M, et al. Dynamin A, Myosin IB and Abp1 couple phagosome maturation to F-actin binding. Traffic. 2012;13:120–30.

3. Hurley JH. ESCRTs are everywhere. The EMBO Journal. 2015;34:2398–407.

4. Mattei S, Klein G, Satre M, Aubry L. Trafficking and developmental signaling: Alix at the crossroads. European Journal of Cell Biology. 2006;85:925–36.

5. Okumura M, Ichioka F, Kobayashi R, Suzuki H, Yoshida H, Shibata H, et al. Penta-EF-hand protein ALG-2 functions as a Ca2+-dependent adaptor that bridges Alix and TSG101. Biochem Biophys Res Commun. 2009;386:237–41.

6. Lee SM, Chin L-S, Li L. Charcot-Marie-Tooth disease-linked protein SIMPLE functions with the ESCRT machinery in endosomal trafficking. J Cell Biol. 2012;199:799–816.

7. López-Jiménez AT, Cardenal-Muñoz E, Leuba F, Gerstenmaier L, Barisch C, Hagedorn M, et al. The ESCRT and autophagy machineries cooperate to repair ESX-1-dependent damage at the Mycobacterium-containing vacuole but have opposite impact on containing the infection. PLoS Pathog. 2018;14:e1007501.

8. Portal-Celhay C, Tufariello JM, Srivastava S, Zahra A, Klevorn T, Grace PS, et al. Mycobacterium tuberculosis EsxH inhibits ESCRT-dependent CD4+ T-cell activation. Nat Microbiol. 2016;2:16232.

9. Mehra A, Zahra A, Thompson V, Sirisaengtaksin N, Wells A, Porto M, et al. Mycobacterium tuberculosis type VII secreted effector EsxH targets host ESCRT to impair trafficking. PLoS Pathog. 2013;9:e1003734.

10. Hagedorn M, Rohde KH, Russell DG, Soldati T. Infection by Tubercular Mycobacteria Is Spread by Nonlytic Ejection from Their Amoeba Hosts. Science. 2009;323:1729–33.

11. Hagedorn M, Soldati T. Flotillin and RacH modulate the intracellular immunity of Dictyostelium to Mycobacterium marinum infection. Cellular Microbiology. 2007;9:2716–33.

12. Gerstenmaier L, Pilla R, Herrmann L, Herrmann H, Prado M, Villafano GJ, et al. The autophagic machinery ensures nonlytic transmission of mycobacteria. Proceedings of the National Academy of Sciences. 2015;112:E687–92.

13. Cardenal-Muñoz E, Arafah S, López-Jiménez AT, Kicka S, Falaise A, Bach F, et al. Mycobacterium marinum antagonistically induces an autophagic response while repressing the autophagic flux in a TORC1- and ESX-1-dependent manner. PLoS Pathog. 2017;13:e1006344.

14. Mesquita A, Cardenal-Muñoz E, Dominguez E, Muñoz-Braceras S, Nuñez-Corcuera B, Phillips BA, et al. Autophagy in Dictyostelium: Mechanisms, regulation and disease in a simple biomedical model. Autophagy. 2017;13:24–40.

15. Ravanel K, de Chassey B, Cornillon S, Benghezal M, Zulianello L, Gebbie L, et al. Membrane sorting in the endocytic and phagocytic pathway of Dictyostelium discoideum. European Journal of Cell Biology. 2001;80:754–64.

16. Zhang X, Zhuchenko O, Kuspa A, Soldati T. Social amoebae trap and kill bacteria by casting DNA nets. Nat Commun. 2016;7:10938.

17. Li Z, Dugan AS, Bloomfield G, Skelton J, Ivens A, Losick V, et al. The amoebal MAP kinase response to Legionella pneumophila is regulated by DupA. Cell Host Microbe. 2009;6:253–67.

18. Carr AC, Maggini S. Vitamin C and Immune Function. Nutrients. 2017;9.

19. Wienke D, Drengk A, Schmauch C, Jenne N, Maniak M. Vacuolin, a flotillin/reggie-related protein from Dictyostelium oligomerizes for endosome association. Eur J Cell Biol. 2006;85:991–1000.

20. Fey P, Dodson RJ, Basu S, Chisholm RL. One stop shop for everything Dictyostelium: dictyBase and the Dicty Stock Center in 2012. Methods Mol Biol. 2013;983:59–92.
